# Phlebotomine sand flies from Madagascar (Diptera: Psychodidae). VII. An identification key for *Phlebotomus* with the description of *Phlebotomus* (*Anaphlebotomus*) *vaomalalae* n. sp.

**DOI:** 10.1051/parasite/2013005

**Published:** 2013-02-19

**Authors:** Fano José Randrianambinintsoa, Nicole Léger, Vincent Robert, Jérôme Depaquit

**Affiliations:** 1 Université de Reims Champagne-Ardenne, ANSES, EA4688 – USC « Transmission vectorielle et épidémiosurveillance de maladies parasitaires (VECPAR) » 51 rue Cognacq-Jay 51096 Reims Cedex France; 2 Département de Biologie Animale, Faculté des Sciences, Université d’Antananarivo Antananarivo Madagascar; 3 MIVEGEC, UMR IRD 224-CNRS 5290-UM1-UM2 911 avenue Agropolis BP 64501 34394 Montpellier Cedex 5 France; 4 63 avenue Pierre Sémart 94210 La Varenne Saint Hilaire France

**Keywords:** Phlebotomine sand flies, *Anaphlebotomus*, Madagascar, morphology, molecular taxonomy

## Abstract

An identification key of the *Phlebotomus* in Madagascar is proposed as well as the description of the male and female *Phlebotomus* (*Anaphlebotomus*) *vaomalalae* n. sp. from Mikea Forest in the south-west of Madagascar. The assignation of this new species to the genus *Phlebotomus* is based on the presence of mesanepisternal setae. Its inclusion in the subgenus *Anaphlebotomus* is based on the males on the presence of four spines on the style, the lack of a coxite basal process and the existence of a bifurcated paramere. The female has cibarial and pharyngeal armature and spermathecal architecture similar to *Phlebotomus fertei* and *Phlebotomus berentiensis*, two other Malagasy species which belong to *Anaphlebotomus.* Male and female are held to belong to the same species because of their morphological characters, the homology (100%) of their partial cytochrome b mtDNA sequences and their capture in the same trap. *P. vaomalalae* n. sp. is a small species compared to the other *Phlebotomus* species of Madagascar. The cibarium of the male and the female of *P. vaomalalae* n. sp. is armed with teeth, like those of other Malagasy *Phlebotomus*. However, it differs in the arrangement and shape of the respective teeth and denticles. The male of *P. vaomalalae* n. sp. looks like that of *P. fontenillei* due to its tuft of coxal setae (lacking in *P. berentiensis* and *P. fertei*) but differs from this species by the location of this tuft. As *P. fertei* and *P. berentiensis,* there is no spermathecal common duct in *P. vaomalalae* n. sp.

## Abstract (fr)

Une clé d’identification des *Phlebotomus* de Madagascar est proposée ainsi que la description du mâle et de la femelle de *Phlebotomus* (*Anaphlebotomus*) *vaomalalae* n. sp. provenant de la Forêt de Mikea dans le sud-ouest de Madagascar. Le rattachement de cette nouvelle espèce au genre *Phlebotomus* repose sur la présence de soies mésanepisternales. Son rattachement au sous-genre *Anaphlebotomus* repose chez le mâle sur la présence de quatre épines sur le style, l’absence de lobe basal du coxite et l’existence d’un paramère bilobé. La femelle possède des armatures pharyngienne et cibariale, ainsi que des spermathèques dont les architectures sont comparables à celles de deux autres espèces (*Phlebotomus fertei* et *Phlebotomus berentiensis*) connues de Madagascar et appartenant à *Anaphlebotomus*. L’assignation du mâle et de la femelle à la même espèce est fondée sur leurs caractères morphologiques, sur l’homologie (100 %) de leurs séquences d’une partie du cytochrome b de l’ADN mitochondrial, et sur leur capture réalisée dans le même piège. *P. vaomalalae* n. sp. est une espèce de petite taille par rapport aux autres *Phlebotomus* de Madagascar. Le cibarium du mâle et de la femelle de *P. vaomalalae* n. sp. est armé de dents, comme ceux des autres espèces malgaches. Il en diffère néanmoins par la disposition et la forme respective des dents et des denticules. Le mâle de *P. vaomalalae* n. sp. est proche de celui de *P. fontenillei* en raison du partage d’une touffe de soies coxales (absente chez *P. berentiensis* et *P. fertei)*, mais diffère de cette espèce par la position de cette touffe. Comme chez *P. fertei* et *P. berentiensis*, les spermathèques de *P. vaomalalae* n. sp. ne présentent pas de conduit commun.

## Introduction

The first record of *Phlebotomus* Rondani & Berté in Madagascar was reported in 2002 when Depaquit *et al.* [1] described the male of *Phlebotomus fertei* Depaquit, Léger & Robert and the female of *Phlebotomus huberti* Depaquit, Léger & Robert. Subsequently, the same authors described the female of *P. fertei* and the male of *Phlebotomus berentiensis* (Léger & Rodhain, 1978) [2]. They also included the latter species [3] in the genus *Phlebotomus* [2]. Later, they described *P. fontenillei* Depaquit, Léger & Robert whose female remains unknown [4]. Therefore, before the present study, the fauna of *Phlebotomus* from Madagascar included four species: *P. berentiensis*, *P. fertei, P. huberti* and *P. fontenillei*. In the archipelago of Comoros, no species of the genus *Phlebotomus* has been recorded [5].

Here, we describe the male and the female of a new species from the Southwest of Madagascar: *Phlebotomus* (*Anaphlebotomus*) *vaomalalae* n. sp. According to the increasing number of species, an identification key is provided for the identification of *Phlebotomus* from Madagascar (males and females).

## Materials and methods

### Study site and collection method

The two sand flies examined (one male and one female) were collected in the southwestern administrative region (ex-province of Toliara), in the forest of Mikea, a dry deciduous forest belonging to the western phytogeographical region [6].

Sand flies were caught using CDC miniature light traps at the site named Abrahama – Jiloriaky, 7.5 km north-east of Tsifota (22° 48.0′ S – 43° 26.0′ E) and 60 m a.s.l., over five consecutive nights from February 21 to 25, 2003. These collections were carried out in lowland forest, dominated by trees (Didieraceae, baobab trees and lianas) reaching 15 m height, in high thickets on red to whitish rich alluvial, sandy soil. The forest is partly affected by anthropogenic pressure except where the undergrowth is impenetrable.

### Morphological analysis

The sand flies collected were stored in 96% ethanol. The head and genitalia were cut off in a drop of ethanol, cleared in boiling Marc-André solution and mounted between slide and cover slide for species identification. The body related to the specimen was dried and stored in a vial at −20 °C before DNA extraction. The specimens were observed under a BX50 microscope and measured using the Perfect Image software (Aries Company, Chatillon, France) and a video camera connected to the microscope. Drawings were made using the camera lucida installed on the microscope. To allow long-term preservation of the specimens, they were remounted on slides in Canada balsam, after complete processing by washing, dehydration in baths of ethanol 70–100 and immersion in creosote.

### Molecular analysis

Genomic DNA was extracted from the thorax, wings, legs and abdomen of individual sand flies using the QIAmp DNA Mini Kit (Qiagen, Germany) following the manufacturer’s instructions, modified by crushing the sand fly tissues with a piston pellet (Treff, Switzerland), and using an elution volume of 200 μL, as detailed in Depaquit *et al.* [2]. All the mtDNA amplifications were performed in a 50 μL volume using 5 μL of extracted DNA solution and 50 pmol of each of the primers. The PCR mix contained (final concentrations) 10 mM Tris-HCl (pH 8.3), 1.5 mM MgCl_2_, 50 mM KCl, 0.01% Triton X 100, 200 μM dNTP each base and 1.25 units of 5 prime Taq polymerase (Eppendorf, Germany). The cycle begins with an initial denaturation step at 94 °C for 3 min and finishes with a final extension at 68 °C for 10 min. Amplification of a fragment of cytochrome B gene was undertaken using the primers N1N-PDR and C3B-PDR [7]. Amplicons were analysed by electrophoresis in 1.5% agarose gel containing ethidium bromide. Direct sequencing in both directions was performed using the primers used for DNA amplification. The correction of sequences was done using the Pregap and Gap software included in the Staden Package [8].

## *Phlebotomus vaomalalae* Randrianambinintsoa, Léger & Depaquit n. sp.

Genus *Phlebotomus* Rondani & Berté, in Rondani, 1840

Subgenus *Anaphlebotomus* Theodor, 1948

urn:lsid:zoobank.org:act:7C7E487F-00D4-4F04-AD89-7DCCB 9454700

GenBank Accession Nos.: male holotype, JX512360; female paratype JX512361.

Type-locality: Abrahama-Jiloriaky, forest of Mikea, Madagascar (22° 48.0′ S, 43° 26.0′ E).

Type-material: holotype and 1 paratype (allotype), Collection of Entomology, Muséum National d’Histoire Naturelle, Paris (MNHN), date February 21–25, 2003.

Etymology: We dedicate this species to Dr. Vaomalala Raharimanga, epidemiologist at the Institut Pasteur de Madagascar, who kindly collected specimens described in this work.

Authorship: Note that the authors of the new taxon are different from the authors of this paper; Article 50.1 and Recommendation 50A of International Code of Zoological Nomenclature [10].

### Description (male: Figure 1; female: Figure 2)

The terminology used in the description below is that of Abonnenc [9].

**Figure 1.**
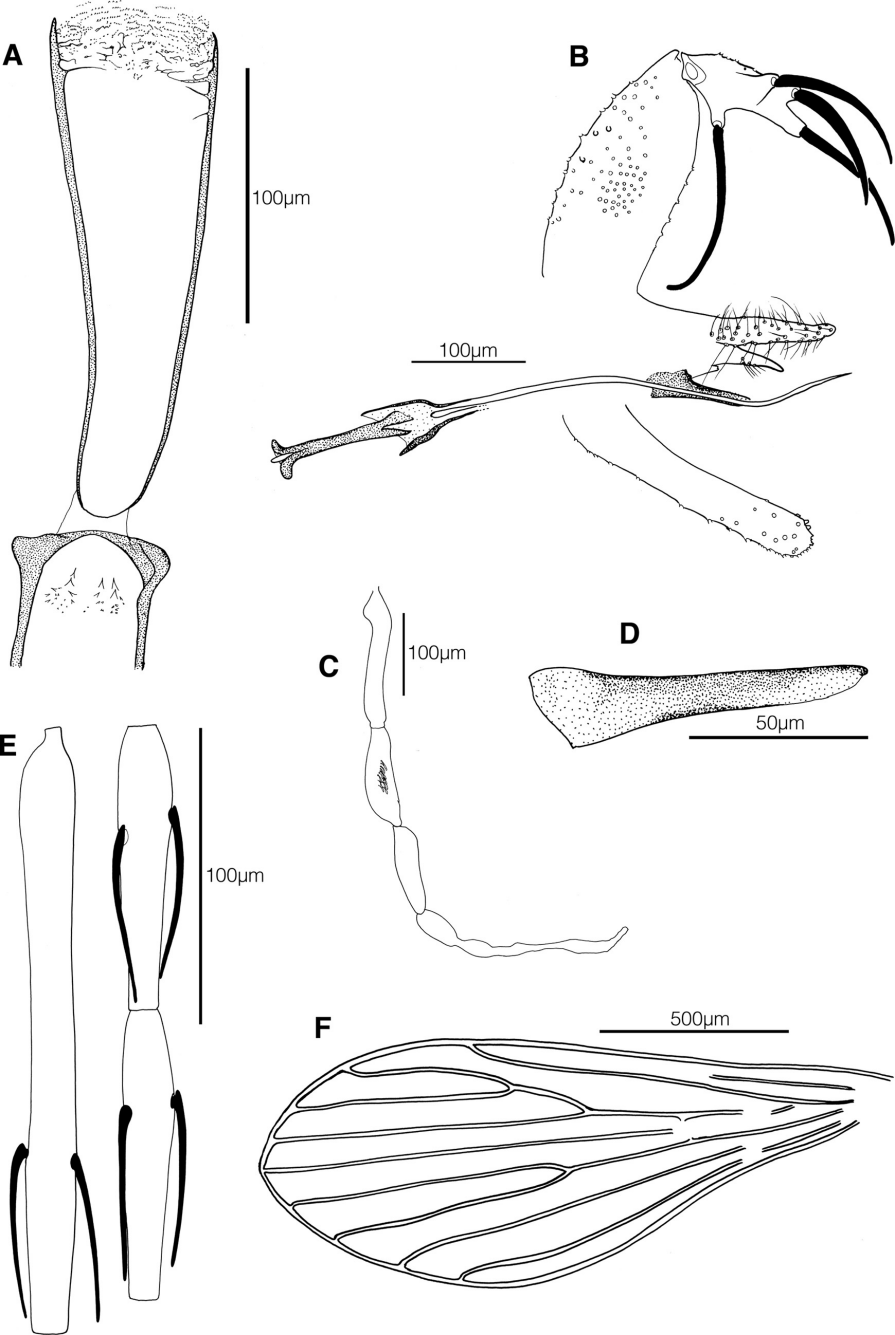
*Phlebotomus* (*Anaphlebotomus*) *vaomalalae* n. sp. male. A, pharynx and cibarium; B, genitalia; C, palp; D, aedeagus; E, antennal segments III, IV et V; F, wing.

**Figure 2.**
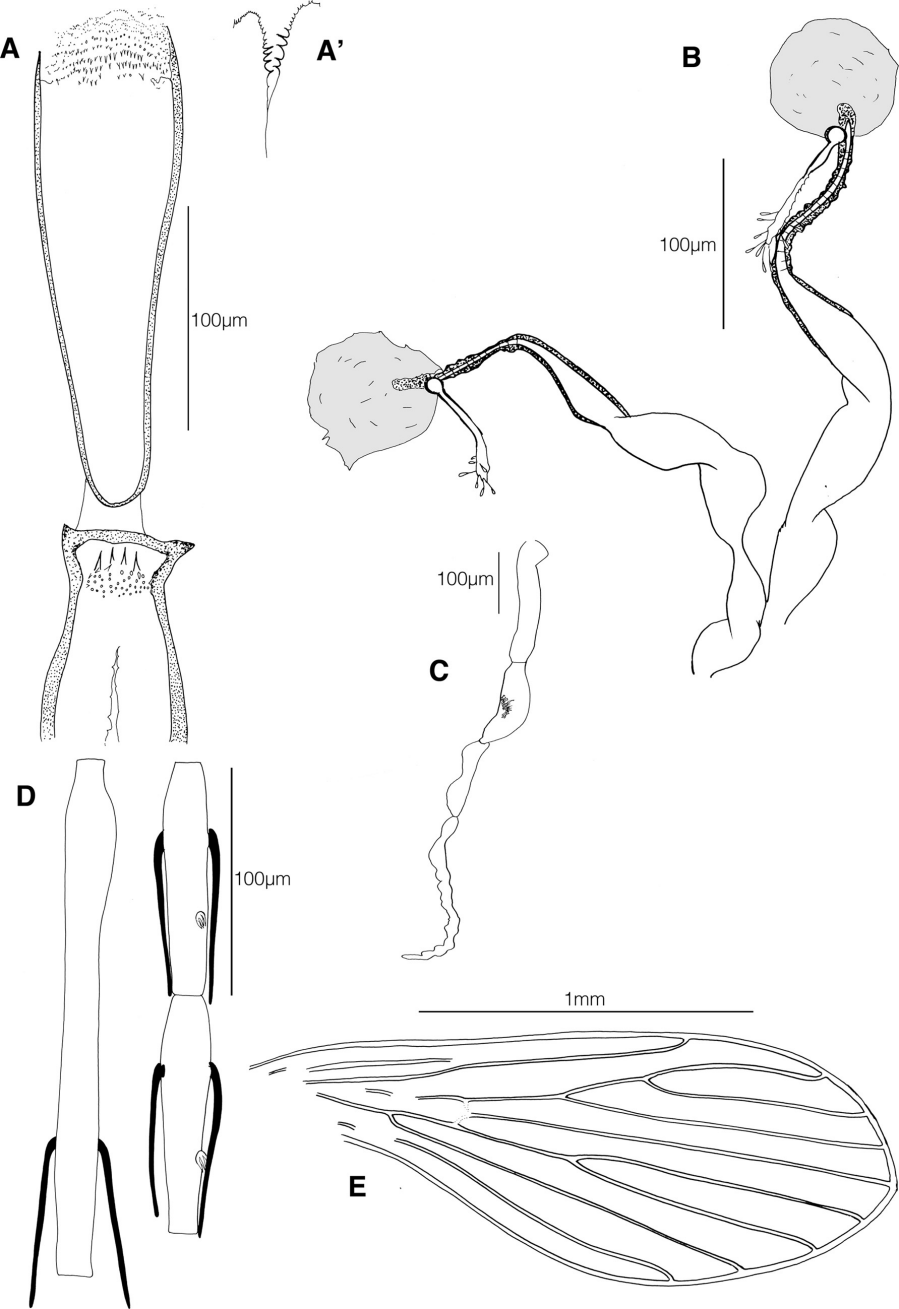
*Phlebotomus* (*Anaphlebotomus*) *vaomalalae* n. sp. female. A, pharynx and cibarium; A′, a view of the posterior part of the pharyngeal armature; B, spermathecae; C, palp; D, antennal segments III, IV and V; E, wing.

#### Male

Holotype.

***Head*.** Interocular suture: incomplete. Cibarial armature with some posterior teeth directed backwards and some anterior denticles. Pharynx quite narrow, with a discrete armature composed of very small aligned teeth, forming ripples. Some well-developed lateral teeth in the anterior part of the pharynx. Palpal formula: 1, 4, 3, 2, 5. A few Newstead’s scales in a patch on mesal face of segment 3. Antennal formula: 2/III–XII... (next segments were missing) with long ascoids, not reaching the next article. AIII = 203 μm less than (AIV + AV). Labrum = 223 μm. AIII/E = 0.91.

***Thorax*.** Four setae on the lower mesanepisternum not observable after the remounting in Canada balsam. Wing: length = 1663 μm, width = 601 μm, *α* = 433 μm, *β* = 221 μm, *δ* = 89 μm, *γ* = 221 μm, *π* = 44 μm. Width/*γ* ratio = 2.72.

***Genital armature*.** Coxite: length = 216 μm bearing a tuft of 35 setae located on the central part of its internal face. Style: length = 128 μm, narrow, with four spines: one at the top carried by a peduncle, one lower external on the basal part and the two inserted between them. Forked paramere: Upper lobe part exhibiting many setae. Lower lobe thin and shorter, showing a group of six setae on its lower side. Surstyle: length = 223 μm. Aedeagus: length = 61 μm, straight, regularly tapering towards the distal end. Genital filaments: length = 376 μm, isodiametric. Genital pump = 151 μm. Genital filaments/pump = 2.41.

#### Female

Paratype (allotype)

***Head*.** Interocular suture incomplete. Cibarium armed with four vertical teeth oriented backwards and more than 30 anterior denticles. Pharynx widens evenly towards the rear. Small pharyngeal armature containing small dots-like teeth at the back and short anterior teeth organized in rows. Palpal formula: 1, 4, 3, 2, 5. A few Newstead’s scales in a patch on mesal face of segment 3. Antennal formula: 2/III–XV with long ascoids reaching and sometimes exceeding the next articulation. AIII = 221 μm, less than (AIV + AV). Labrum = 304 μm. AIII/E = 0.73.

***Thorax*.** Mesanepisternal setae not observable due to the mounting. Wing: length = 1939 μm, width = 699 μm, *α* = 535 μm, *β* = 241 μm, *δ* = 108 μm, *γ* = 260 μm, *π* = 64 μm. Width/*γ* = 2.69.

***Spermathecae*.** The body of each spermatheca consists of two successive parts. The apical section is larger (diameter = 34 μm), and is bordered by a thin and irregular wall. The smaller (diameter = 5 μm) is sclerotized and bounded by a thick rigid wall (diameter = 5 μm). The neck is 30 μm long and carries the head of the spermatheca. Absence of common duct is noticed. Individual ducts are approximately 180 μm long and tapered. In their apical part, they are narrow and their walls thicken irregularly and reveal discreet rings. Furca: observation difficult on our specimen.

## Discussion

The presence of lower mesanepisternal setae justifies the inclusion of the new species in the genus *Phlebotomus*.

Until the revision of the subgenus *Anaphlebotomus*, suggested by Depaquit *et al.* [2, 4] is undertaken, we have classified *P. vaomalalae* n. sp. in this subgenus due to the presence of the male characters listed by Theodor [11] when creating the subgenus: style bearing four spines absence of basal process on the coxite, and presence of a forked paramere. The female characters listed by that author for *Anaphlebotomus* do not justify the inclusion of *P. vaomalalae* n. sp. in this subgenus, although *P. vaomalalae* n. sp. should obviously be grouped with two other *Phlebotomus* females of the subgenus *Anaphlebotomus* already described from Madagascar: *P. fertei* and *P. berentiensis*. The females of these three species share a similar architecture of the spermathecae and similar pharyngeal and cibarial armatures. They differ markedly from the female of *P. huberti* that presents ringed frame spermathecae and a highly developed pharyngeal armature. The status of the latter species deserves special attention in light of the future description of the male.

We associate the male and female specimens described here with the following arguments. They were captured in the same location and in the same set of capture in light traps. The two specimens taken are the only *Phlebotomus* collected (along with 40 sand flies of the genus *Grassomyia*) in these series of catches. The two sexes are both close to *P. fertei* and *P. berentiensis*. Sequences of the Cytochrome b gene are identical (100% homology).

To date, four species belonging to the subgenus *Anaphlebotomus* have been reported in Madagascar.

### Differential diagnosis

*P. vaomalalae* n. sp. is smaller than other *Phlebotomus* species of Madagascar.

In males (Table 1) and females (Table 2), the third antennal segment is much shorter in *P. vaomalalae* n. sp. than in *P. fertei* and slightly shorter than other Malagasy *Phlebotomus*, although the small sample size (only one specimen observed for each sex) does not allow us to assert it categorically. AIII is shorter than the length of the labrum in *P. vaomalalae* n. sp. whereas in the other species of *Phlebotomus* from Madagascar, AIII is longer than the labrum.

**Table 1.** Male measurements (in μm).

	*P. vaomalalae* n. sp.	*P. fertei* [1]	*P. berentiensis* [2]	*P. fontenillei* [4]
*Head*				
AIII	203	424 (394–454)	226	235
AIV	90	174 (160–188)	101	112.5
AV	94	174.5 (164–185)	103	112.5
Labrum	223	288 (273–303)	182	210
AIV + AV	184	348.5 (162–186.5)	204	225
Antennal formula	2/III–XII*	2/III–XV	2/III–XII	2/III–XII
Palpal formula	1, 4, 3, 2, 5	1, 4, 3, 2, 5	1, 4, (2,3), 5	1, 4, (3, 2), 5
*Wings*				
Length	1,663	2,195 (2,140–2,250)	1,840	1,720
Width	601	600 (570–630)	605	600
*α*	433	565 (500–630)	470	410
*β*	221	250 (230–270)	225	280
*δ*	89	210 (170–250)	131	70
*γ*	221	195 (+170–220)	261	244
*π*	44	90 (+70–110)	83	100
w/γ	2.72	3.08	2.32	2.46
*Genitalia*				
Style length	128	140 (135–145)	125	140
Insertion of the most basal setae of the style	Basal	Median	Basal	Basal
Coxite length	216	242.5 (235–250)	230	250
Number of coxal setae	35 tufted	10–15	10	40–45 tufted
Aedeagus	61	105 (90–120)	90	90
Surstyle	223	260 (250–270)	200	
Genital filaments length	376	620 (600–640)	315	435
Genital pump length	151	105 (90–120)	167	170

* Antenna not complete.

**Table 2.** Female measurements (in μm).

	*P. vaomalalae* n. sp.	*P. fertei* [2]	*P. berentiensis* [2]	*P. huberti* [1]
	(*n* = 1)	(*n* = 20)	(*n* = 1)	(*n* = 2)
*Head*				
AIII	221	401 (332–483)	227	260
AIV	106	173 (146–224)	104	125
AV	102	171 (145–216)	98	125
Labrum	304	447 (379–540)	153	190 (180–200)
AIV + AV	208	344	202	250
Antennal formula	2/III–XV	2/III–XV	2/III–XV	2/III–XV
Palpal formula	1, 4, 3, 2, 5	1, 4, 3, 2, 5	1, 4, 3, 2, 5	1, 2, 3, 4, 5
*Wings*				
Length	1,939	2,396 (2,022–3,017)	2,041	1,730
Width	699	676 (544–902)	645	450
*α*	535	658 (480–994)	548	370
*β*	241	303 (232–405)	279	330
*δ*	108	218 (88–418)	164	220
*γ*	260	240 (191–307	282	250
*π*	64	99 (42–165)	95	120
w/γ	2.69	2.8	2.29	1.83
*Spermathecae*				
Ducts	182	323 (237–480)	162	470

The wings of *P. vaomalalae* n. sp. are shorter than those of *P. fertei*. Their measurements are similar to those of other species of *Phlebotomus* from Madagascar. The *π* (pi) of *P. vaomalalae* n. sp. is much lower than that of the other *Phlebotomus*, in both sexes (Tables 1 and 2).

The cibarium of the male and the female of *P. vaomalalae* n. sp. is armed with teeth, like those of other Malagasy *Phlebotomus*. However, it differs in the arrangement and shape of the respective teeth and denticles. The cibarial armature of the male of *P. vaomalalae* n. sp. presents eight well-marked teeth with a few denticles. It is more developed in the male of *P. fontenillei* [2, 4]. In contrast, the female of *P. vaomalalae* n. sp. carries only four teeth and thirty denticles. It is also clearly marked in *P. fertei* female and *P. berentiensis* [2, 4].

The pharyngeal armature of *P. vaomalalae* n. sp. consists of small teeth and denticles irregularly organized in multiple rows.The pharyngeal armature of the male differs from that of *P. fertei*, which is formed of small teeth disposed on seven or eight concentric circular arcs, from that of *P. berentiensis*, which is narrow and lined with small pointed teeth and lateral teeth, and from that of *P. fontenillei*, which is composed of tapered corrugations and is well defined with the presence of some front lateral teeth [2, 4].

The female of *P. vaomalalae* n. sp. differs from that of *P. huberti* by the armature of the pharynx and its spermathecal body with no ring. Its pharyngeal and cibarial armatures are comparable with those of *P. berentiensis* and *P. fertei*. They differ in the number, shape and arrangement of teeth and denticles. The pharyngeal armature of *P. fertei* and *P. fontenillei* comprises lateral teeth not present in *P. berentiensis* and *P. vaomalalae* n. sp.

The coxite and aedeagus of *P. vaomalalae* n. sp. are shorter than those of other *Phlebotomus* (Table 1).

The male of *P. vaomalalae* n. sp. looks like that of *P. fontenillei* due to its tuft of coxal setae, a tuft which is lacking in *P. berentiensis* and *P. fertei*. This tuft is implanted in the middle part of the inner face of coxite in *P. vaomalalae* n. sp. while it is located on the bottom part of the inner face of the coxite in *P. fontenillei*.

The spermathecae of *P. vaomalalae* n. sp. present no common duct, as in *P. fertei* and *P. berentiensis*. These species differ by spermathecal duct length, the structure of the tip, the basal sclerotization observed in *P. fertei* and the structure and thickness of the successive chambers, especially the largest one (Table 2).

### Identification key of the males of *Phlebotomus* of Madagascar

1. Presence of a tuft of setae on the coxite ……… 2
  Absence of a tuft of setae on the coxite ……… 3
2. A tuft of over 40 setae located in the lowest part of the inner face of coxite ……… *P. fontenillei*
  A tuft of 35 setae located in the middle portion of the inner face of coxite ……… *P. vaomalalae* n. sp.
3. Short and isodiametric genital filaments ……… *P. berentiensis*
  Long and non-isodiametric genital filaments ……… *P. fertei*

### Identification key of the females of *Phlebotomus* of Madagascar

1. Spermathecae ringed ……… *P. huberti*
  Spermathecae not ringed ……… 2
2. Largest spermathecal chamber with thick wall ……… *P. berentiensis*
  Largest spermathecal chamber with thin wall ……… 3
3. Spermathecal ducts long and sclerotized at their base ……… *P. fertei*
  Spermathecal ducts short and not sclerotized at their base ……… *P. vaomalalae* n. sp

## References

[1] Depaquit J, Léger N, Robert V. 2002 Première mention de *Phlebotomus* à Madagascar (Diptera : Psychodidae). Description de *Phlebotomus* (*Anaphlebotomus*) *fertei* n. sp. et de *Phlebotomus* (*Anaphlebotomus*) *huberti* n. sp. Parasite, 9, 325–331 12514946 10.1051/parasite/2002094325

[2] Depaquit J, Léger N, Ferté H, Robert V. 2004 Les Phlébotomes de Madagascar (Diptera – Psychodidae). II – Description de la femelle de *Phlebotomus* (*Anaphlebotomus*) *fertei* Depaquit, Léger & Robert, 2002; description du mâle et redescription de la femelle de *Phlebotomus* (*Anaphlebotomus*) *berentiensis* (Léger & Rodhain, 1978) comb. nov. Parasite, 11, 201–209 15224582 10.1051/parasite/2004112201

[3] Léger N, Rodhain F. 1978 *Sergentomyia berentiensis* n. sp. (Diptera, Psychodidae). Description à partir d’un exemplaire femelle récolté à Madagascar. Bulletin de la Société de Pathologie Exotique, 71, 476–479 755540

[4] Depaquit J, Léger N, Robert V. 2004 Les Phlébotomes de Madagascar. III – Description de *Phlebotomus* (*Anaphlebotomus*) *fontenillei* n. sp. Parasite, 11, 261–265 15490749 10.1051/parasite/2004113261

[5] Randrianambinintsoa FJ, Depaquit J, Brengues C, Dhondt C, Yahaya I, Ouledi A, Léger N, Robert V. 2012 First record of Phlebotomine sand flies (Diptera: Psychodidae) in the Comoros Archipelago with description of *Sergentomyia* (*Vattieromyia*) *pessoni* n. sp. and *S*. (*Rondanomyia*) *goodmani comorensis* n. ssp. Parasite, 19, 195–206 22910662 10.1051/parasite/2012193195 PMC3671448

[6] Humbert H. 1955 Les Territoires phytogéographiques de Madagascar. Leur cartographie. Colloque sur les Régions écologiques du Globe, Paris 1954. Annales de Biologie (Paris), 31, 195–204

[7] Esseghir S., Ready P-D, Killick-Kendrick R, Ben Ismail R. 1997 Mitochondrial haplotypes and phylogeography of *Phlebotomus* vectors of *Leishmania major*. Insect Molecular Biology. 6, 225–225 10.1046/j.1365-2583.1997.00175.x 9272439

[8] Bonfield JK, Staden R. 1996 Experiment files and their application during large-scale sequencing projects. DNA Sequencing, 6, 109–117 10.3109/10425179609010197 8907307

[9] Abonnenc E. 1972 Les phlébotomes de la région éthiopienne (Diptera, Psychodidae). Cahiers ORSTOM, Série Entomologie Médicale et Parasitologie, 55, 1–239

[10] International Code of Zoological Nomenclature 1999 The International Trust for Zoological Nomenclature (ed.), London

[11] Theodor O. 1948 Classification of the Old World species of the subfamily *Phlebotominae* (*Diptera*: Psychodidae). Bulletin of Entomological Research, 39, 85–118 18865548 10.1017/s0007485300024305

